# Assessment of Microvascular Function Based on Flowmotion Monitored by the Flow-Mediated Skin Fluorescence Technique

**DOI:** 10.3390/bios14100459

**Published:** 2024-09-25

**Authors:** Andrzej Marcinek, Joanna Katarzynska, Katarzyna Cypryk, Agnieszka Los-Stegienta, Jolanta Slowikowska-Hilczer, Renata Walczak-Jedrzejowska, Jacek Zielinski, Jerzy Gebicki

**Affiliations:** 1 Institute of Applied Radiation Chemistry, Lodz University of Technology, 90-924 Lodz, Poland; andrzej.marcinek@p.lodz.pl; 2 Angionica Ltd., 90-924 Lodz, Poland; joanna.katarzynska@angionica.com.pl; 3 Department of Internal Diseases and Diabetology, Medical University of Lodz, 92-213 Lodz, Polandagnieszka.los@umed.lodz.pl (A.L.-S.); 4 Department of Andrology and Reproductive Endocrinology, Medical University of Lodz, 92-213 Lodz, Poland; jolanta.slowikowska-hilczer@umed.lodz.pl (J.S.-H.); renata.walczak-jedrzejowska@umed.lodz.pl (R.W.-J.); 5 Department of Athletics, Strength and Conditioning, Poznan University of Physical Education, 61-871 Poznan, Poland; jacekzielinski@wp.pl

**Keywords:** microcirculation, flowmotion, microvascular perfusion, FMSF technique

## Abstract

This review summarizes studies dedicated to the assessment of microvascular function based on microcirculatory oscillations monitored by the Flow-Mediated Skin Fluorescence (FMSF) technique. Two approaches are presented. The first approach uses oscillatory parameters measured under normoxic conditions, expressed as flowmotion (FM), vasomotion (VM), and the normoxia oscillatory index (NOI). These parameters have been used for the identification of impaired microcirculatory oscillations associated with intense physical exercise, post-COVID syndrome, psychological stress, and erectile dysfunction. The second approach involves characterization of the microcirculatory response to hypoxia based on the measurement of hypoxia sensitivity (HS). The HS parameter is used to characterize microvascular complications in diabetes, such as diabetic kidney disease and diabetic foot ulcers. Based on research conducted by the authors of this review, the FMSF parameter ranges characterizing microvascular function are presented. The diagnostic approach to assessing microvascular function based on flowmotion monitored by the FMSF technique has a wide range of applications and the potential to be integrated into widespread medical practice.

## 1. Introduction

Microcirculatory oscillations, known as flowmotion, are a well-recognized feature of blood flow. The mechanistic aspects of this phenomenon have been the subject of extensive research [1,2,3,4]. Flowmotion is regulated by the rhythmic oscillations of blood vessel diameter, known as vasomotion. This term is often used more specifically to characterize vascular tone, caused by oscillations of vascular smooth muscle, and can also be used to describe myogenic microcirculatory oscillations. The major physiological function of flowmotion is to secure the proper perfusion of tissues. Impaired flowmotion can result in vascular resistance and is observed in many diseases and disorders [5,6,7,8,9,10,11,12,13].

So far, the major technique applied for the characterization of flowmotion has been Laser Doppler Flowmetry (LDF) [14,15]. LDF is predominantly applied for the assessment of skin microcirculation. Cutaneous vascular function can also be considered an indicator of general vascular function [16,17]. Analysis of the LDF signal in the frequency domain reveals that microcirculatory oscillations in the low-frequency range (<0.15 Hz) fit into several periodic activities, classified as endothelial (<0.021 Hz), neurogenic (0.021–0.052 Hz), and myogenic (0.052–0.15 Hz) [18,19]. Due to the significant noise associated with LDF measurement, quantitative analysis of the changes in flowmotion associated with different diseases and pathologies is not always possible.

Analysis of flowmotion in the frequency domain can provide fundamental information concerning changes in the blood flow dynamics caused by various diseases. Therefore, there is great demand for a simple and non-invasive diagnostic tool for measuring flowmotion accurately. The recently proposed Flow-Mediated Skin Fluorescence (FMSF) technique appears to meet these requirements [20,21]. The FMSF technique is based on the measurement of nicotinamide adenine dinucleotide (NADH) fluorescence from human skin, predominantly from the epidermis. The epidermis is not vascularized, and the red blood cells do not interfere with the measured NADH fluorescence. In addition, the FMSF technique combined with the post-occlusive reactive hyperemia (PORH) test enables the measurement of the reaction of microcirculatory oscillations to hypoxia. This is important for understanding the function of the microcirculation in diseases associated with ischemia (hypoxia).

This review discusses exemplary cases of perturbed microcirculatory oscillations observed both in healthy individuals and seriously ill patients. Special attention is given to diagnostic aspects of the measured microcirculatory oscillations, in order to understand their role in preventing microvascular perfusion, which is necessary for proper tissue functioning. In some selected cases, the discussion also focuses on the effects of drugs for reversing impaired microvascular function due to disturbed flowmotion.

## 2. Precise Monitoring of Microcirculatory Oscillations Using the FMSF Technique

**Figure 1:**
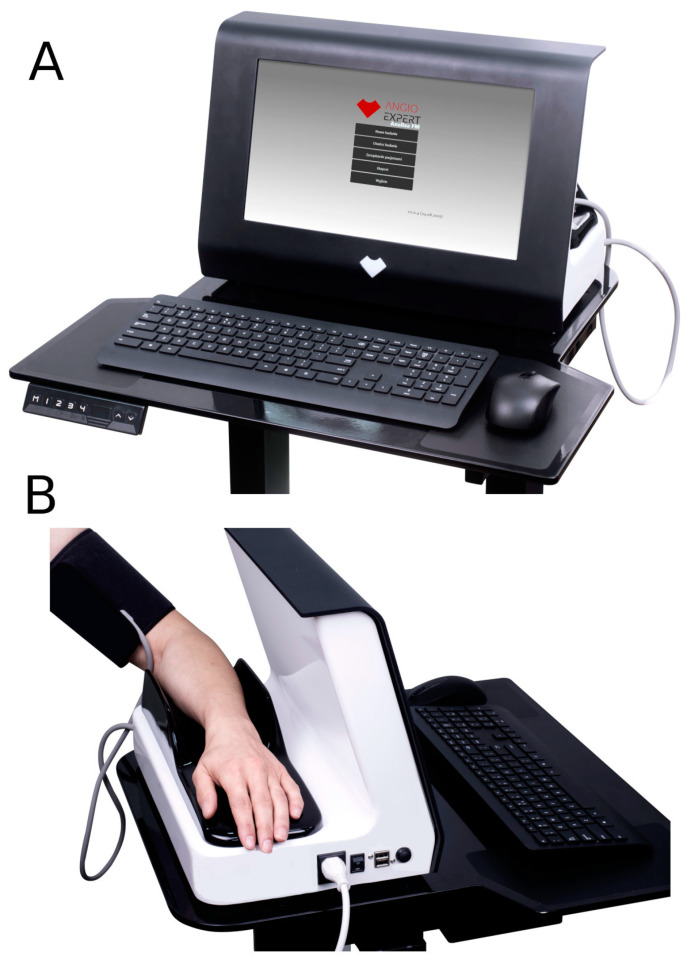
Front (**A**) and back (**B**) views of the AngioExpert device.

The FMSF technique is based on the registered changes in the intensity of NADH fluorescence from the skin on the forearm. These changes are induced by blocking and releasing blood flow in the brachial artery using an occlusion cuff. The measurements are performed using AngioExpert, a diagnostic tool produced by Angionica Ltd. (Lodz, Poland, Figure 1). 

**Figure 2:**
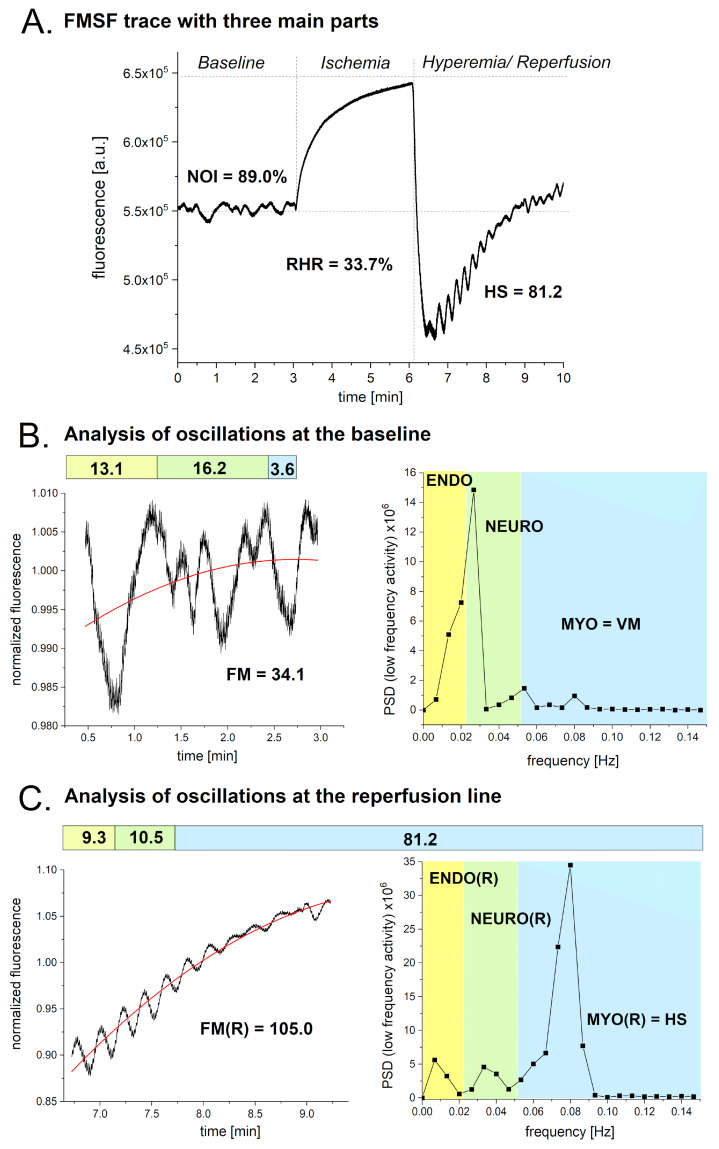
Exemplary FMSF trace recorded using AngioExpert device. (**A**): Visualization of three main parts showing key parameters (RHR, HS, NOI) for each part. (**B**): Analysis of oscillations at baseline. (**C**): Analysis of oscillations at reperfusion line.

An exemplary FMSF trace is presented in Figure 2A. The measurement is divided into three phases. The first phase measures NADH fluorescence at the baseline under normoxic conditions. The second phase, which lasts three minutes, measures ischemia. Finally, the hyperemia/reperfusion phase is initiated by the abrupt release of the pressure in the occlusion cuff. Microcirculatory oscillations are visible on the baseline and the reperfusion line. During the ischemic phase, microcirculatory oscillations are not visible, as blood flow in the forearm is completely blocked. Each part of the FMSF trace is represented by one key parameter. At the baseline, the key parameter is the normoxia oscillatory index (NOI). For the ischemia/hyperemia phase, the key parameter is the Reactive Hyperemia Response (RHR). During the hyperemia/reperfusion phase, the key parameter is hypoxia sensitivity (HS). The procedure for conducting FMSF measurements and the definitions of all parameters are described in detail elsewhere [22,23].

The observed microcirculatory oscillations are shown in Figure 2B (baseline) and Figure 2C (reperfusion line). The frequencies of the oscillations seen in the FMSF signal were analyzed using the Fast Fourier Transform (FFT) algorithm. Periodograms were derived from the FFT of the signal and the Power Spectral Density (PSD) calculated as a mean squared amplitude. The overall intensity of the microcirculatory oscillations observed in the low-frequency domain (<0.15 Hz) at baseline is described by the parameter FM (flowmotion). The value of the FM parameter consists of three components, ENDO (endothelial), NEURO (neurogenic), and MYO (myogenic). Thus, FM = ENDO + NEURO + MYO. Myogenic microcirculatory oscillations are often called vasomotion (VM). Thus, VM = MYO. As the intensities of these parameters are age-dependent, we decided to introduce a new parameter, NOI (normoxia oscillatory index). Thus, NOI = (ENDO + NEURO)/(ENDO + NEURO + MYO) × 100%, which is age-independent and nicely characterizes the relative distribution of the low-frequency oscillations. Similarly, the intensity of the oscillations observed at the reperfusion line can be described as FM(R) = ENDO(R) + NEURO(R) + MYO(R). The intensity of the myogenic microcirculatory oscillations on the reperfusion line is called HS (hypoxia sensitivity). Thus, HS = MYO(R).

In this review, three parameters will be used for the characterization of microcirculatory oscillations at baseline: FM, VM, and NOI. One parameter will be used for the characterization of microcirculatory oscillations on the reperfusion line: HS. To secure a normal distribution, in the statistical analysis, the FM, VM, and HS parameters will often be presented as a logarithm. As microcirculatory oscillations can be measured with very high precision by the FMSF technique, on both the baseline and the reperfusion line, their use for the assessment of microvascular function can be particularly beneficial.

## 3. Selected Examples of Impaired Microcirculatory Oscillations at Normoxia

### 3.1. Intense Physical Exercise

The FMSF technique has been applied for the assessment of the vascular changes associated with intense physical exercise until exhaustion [24,25]. The test is only intended for highly trained athletes. In this study, it was used to verify the body’s reaction to excessive physical overload. As expected, remarkable changes in the FMSF trace were observed just after termination of the test. The baseline level of NADH fluorescence increased, whereas the relative ratio of ischemic to hyperemic responses decreased. These changes clearly demonstrate a shift in the NADH/NAD+ equilibrium towards reduction, indicating oxygen deficiency just after termination of the test. Changes in the microcirculatory oscillations have also been noticed on both the baseline (normoxia) and reperfusion line (hypoxia), but have not been analyzed quantitively.

**Figure 3:**
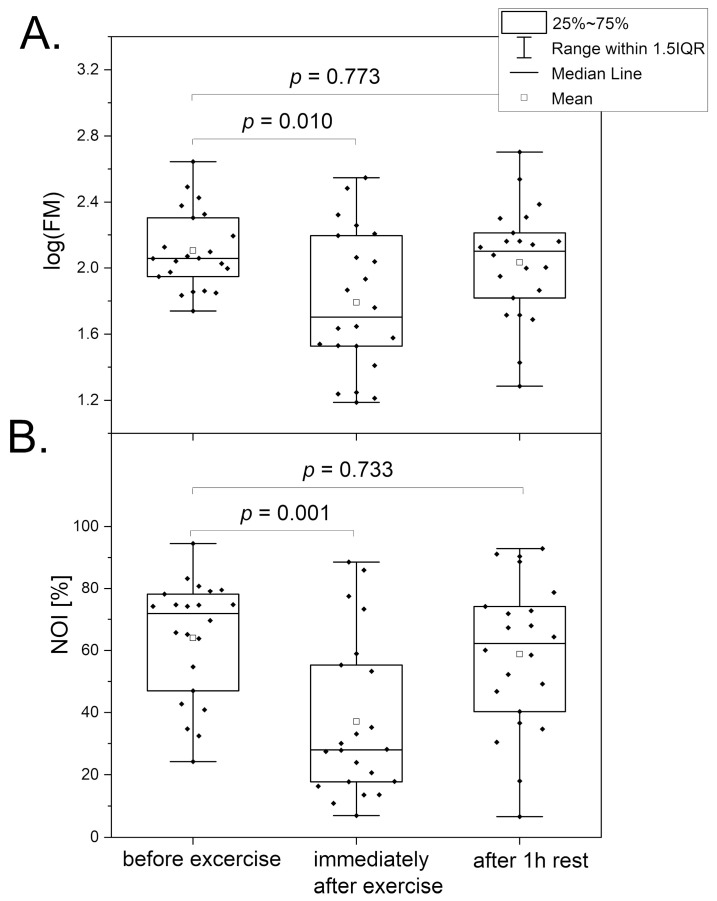
Comparison of the log(FM) (**A**) and NOI (**B**) parameters in a group of highly trained endurance athletes (triathletes—16; long-distance runners—6), n = 22; 15 males; 7 females; mean age 20.0 (16–40 y). Measurements were taken before exercise, after exercise until exhaustion, and after 1 h of rest. The differences between the parameters of the compared groups were considered statistically significant when *p* < 0.05. The *p*-values were calculated by one-way ANOVA with the Tukey post hoc test.

Figure 3 compares the changes in the key parameters (FM and NOI) related to microcirculatory oscillations observed at the baseline. A substantial decrease in the FM and NOI parameters was noted just after termination of the exercise test until exhaustion. Detailed analysis of the observed changes indicated that the intensities of endothelial (ENDO) and neurogenic (NEURO) oscillations were drastically diminished at a relatively preserved intensity of myogenic oscillations (MYO). The FMSF measurements performed after 1 h of rest indicated that in the majority of the athletes, all oscillatory parameters returned to the values observed before initiation of the test. After 3 h of rest, the FMSF oscillatory parameters returned to normal for all tested participants. These observations are not surprising, as it is known that strenuous physical exercise can transiently modify calcium homeostasis, and this effect is persistent until the late recovery phase [26,27]. It is also known that the perturbation of calcium homeostasis strongly affects microcirculatory oscillations [3,4].

The use of the FMSF technique to detect changes in microcirculatory oscillations at baseline does not require the PORH test. Such measurements are fully non-invasive, fast, and accurate. The FMSF technique can be considered a useful tool for monitoring training and for preparing athletes for sports competitions, avoiding overtraining.

### 3.2. Post-COVID Syndrome

**Figure 4:**
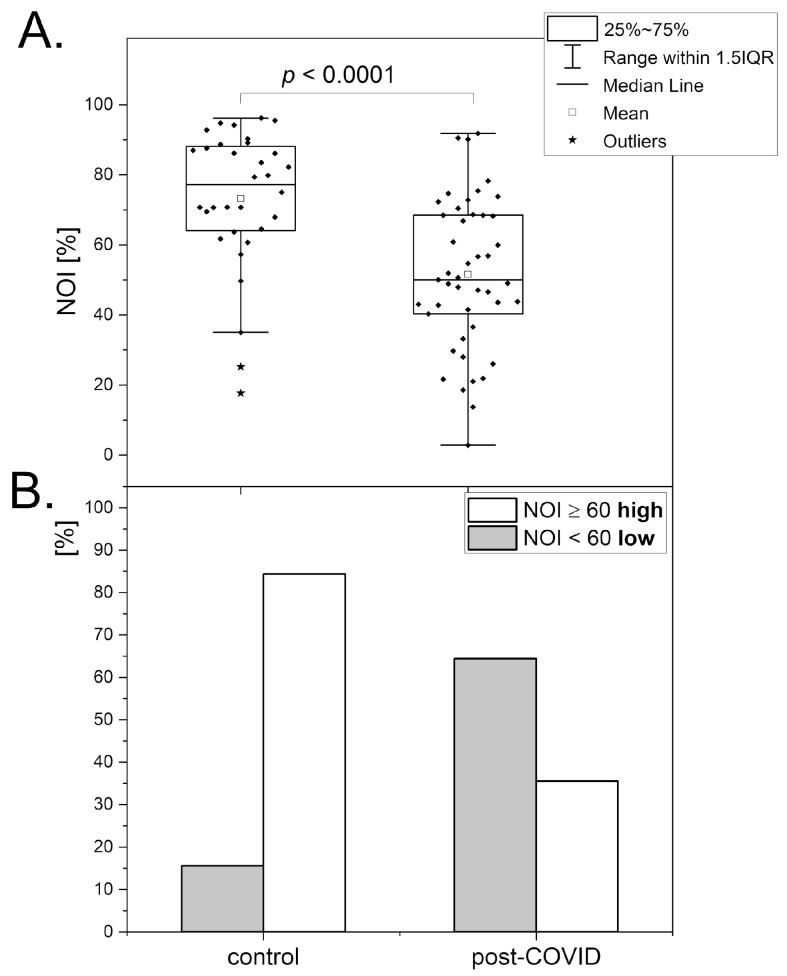
The comparison (**A**) and distribution (**B**) of the NOI parameter in the control group (n = 32, 19 males, 13 females, mean age 37.8 (30–50 y)) and post-COVID group (n = 45, 19 males, 26 females, mean age 41.5 (30–50 y)). The differences between the parameters of the compared groups were considered statistically significant when *p* < 0.05. The *p*-values were calculated from the Mann–Whitney test. Reproduced from [28]. 2022, Dove Medical Press Ltd. Publisher, Auckland, New Zealand.

Chronic fatigue associated with post-COVID syndrome has been studied using the FMSF technique [28]. Both microcirculatory and macrocirculatory parameters were found to be substantially reduced in the post-COVID group compared with the healthy control group. Figure 4 compares both these groups with respect to the NOI parameter and its relative distribution. In fact, the results are very close to those observed for transient fatigue caused by high-intensity exercise presented in the former section. The relative changes in microcirculatory oscillations observed for the post-COVID group vs. the control group and for athletes after vs. before high-intensity exercise are quite similar. However, the mechanistic reasons responsible for the results seem to be quite different, as a full recovery after high-intensity exercise is fast, in contrast to long-lasting post-COVID syndrome. It is likely that the pathophysiology of chronic post-COVID fatigue also originates partly from calcium homeostasis modifications that lead to endothelial dysfunction [29,30]. However, the mechanistic aspects responsible for post-COVID syndrome seem much more complex. A very recent report suggests that post-COVID syndrome may be linked to the dysregulation of metabolic processes in erythrocytes, with associated endothelial and microvascular dysfunction, resulting in reduced oxygen delivery [31].

The chronic fatigue associated with post-COVID syndrome can be evaluated based on microcirculatory oscillations measured by the FMSF technique. This diagnostic approach can be very helpful in monitoring the rehabilitation process. The use of the NOI parameter seems to be particularly appropriate, as it is sufficiently sensitive and also age-independent.

### 3.3. Psychological Stress

**Figure 5:**
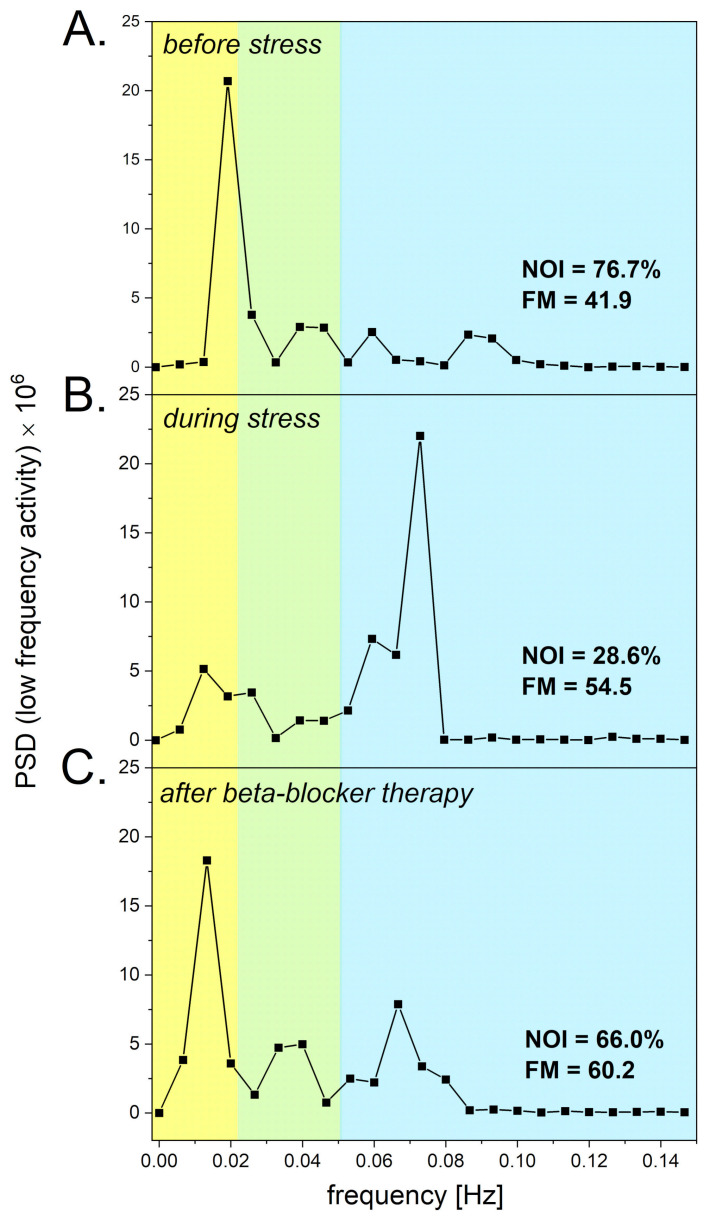
Power Spectral Density (PSD) of the fluorescence signal recorded for a prediabetes patient (male, age 75 y) at the baseline in intervals of endothelial (<0.021 Hz), neurogenic (0.021–0.052 Hz), and myogenic (0.052–0.15 Hz) activity. (**A**): Changes recorded before the appearance of psychological stress. (**B**): Changes observed under prolonged psychological stress. (**C**): changes observed after a week of therapy with a beta-blocker (nebivolol at a daily dose of 1.25 mg). Reproduced from [33]. 2023, Dove Medical Press Ltd. Publisher, Auckland, New Zealand.

It is well known that prolonged psychological stress can be considered a serious risk factor for the function of the entire vascular circulation [32]. Psychological stress can be associated with increased levels of norepinephrine in the circulation, which can also affect peripheral microcirculation and result in microvascular vasoconstriction. We have recently published a paper stating that the effect of psychological stress on the microcirculation can be evaluated using the FMSF technique [33]. Figure 5 shows the Power Spectral Density (PSD) of the fluorescence signal recorded for a patient before stress (segment A), during stress (segment B), and after therapy (segment C). The NOI parameter decreased from 76.7% before stress to 28.6% during psychological stress. The elevated level of norepinephrine due to psychological stress caused microvascular vasoconstriction, resulting in an increased intensity of myogenic microcirculatory oscillations, and consequently, a decrease in the NOI parameter was seen. After treatment with a low dose of beta-blocker, the NOI parameter returned to a value of 66.0%, which is close to the value seen before stress. The results shown in Figure 5 clearly indicate that the NOI parameter seems to be appropriate for the assessment of psychological stress, and that a low dose of beta-blocker can be considered for the treatment of mild psychological stress. It has already been indicated that beta-adrenergic blockade attenuates negative, high-arousal emotions due to psychological stressors [34]. The application of the FMSF technique for the assessment of the vascular consequences of psychological stress appears to be an interesting option, due to the non-invasive nature of the FMSF technique, particularly without use of the PORH test.

### 3.4. Erectile Dysfunction

Erectile dysfunction (ED) is often associated with vascular etiology and is usually the earliest symptom of vascular dysfunction [35,36]. There is growing evidence suggesting an association between ED and cardiovascular diseases (CVD), as well as an increased prevalence of CVD in patients with ED [37,38]. It has been documented that low testosterone levels negatively affect vascular circulation, causing its dysfunction [39,40].

**Figure 6:**
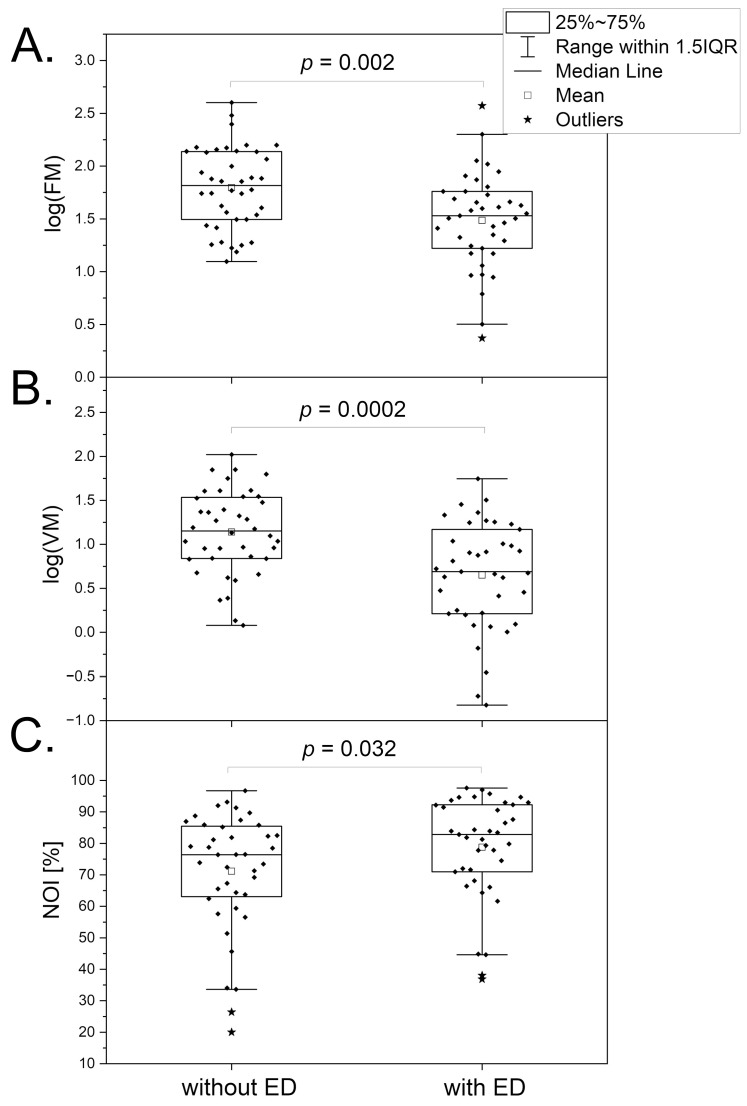
A comparison of the log(FM) (**A**), log(VM) (**B**), and NOI (**C**) parameters for a group of men without erectile dysfunction (ED) (n = 40, mean age 41.2 (24–57 y)) and a group of men with ED (n = 39, mean age 53.3 (27–72 y)). The differences between the parameters of the compared groups were considered statistically significant when *p* < 0.05. The *p*-values were calculated from a two-sample *t*-test for comparisons (**A**,**B**) and the Mann–Whitney test for the comparison (**C**).

Based on rodent models, testosterone can affect myogenic microcirculatory oscillations in testicular arteriolar blood flow, also known as vasomotion (VM) [41,42]. An important hypothesis has been raised regarding whether the effect of testosterone on VM can also be observed in peripheral microcirculation [43]. The results summarized in Figure 6 seem to confirm this hypothesis. It is clearly seen that the low testosterone level linked to ED caused a significant reduction in the FM (segment A) and VM (segment B) parameters and an increase in the value of the NOI parameter (segment C). These results can be interpreted taking into consideration the action of testosterone on endothelial function [44] and calcium homeostasis [45]. There is a known association between testosterone levels and mood disorders [46]. However, this effect can be manifested by an increase in the NOI parameter, which is directly opposed to the observation made for psychological stress, where the NOI parameter decreased due to vasoconstriction caused by norepinephrine. The use of the FMSF technique for the observation of microcirculatory oscillations thus allows for a clear differentiation of psychological stress and stress caused by androgen deficiency. Psychological stress seems to be compensated by an increase in VM in healthy individuals, whereas in individuals with androgen deficiency, this compensatory mechanism may be impaired due to lower VM activity. This compensatory effect is particularly important as chronic psychological stress can result in the development of serious vascular circulatory disorders.

## 4. Evaluation of Microvascular Complications in Diabetes Based on Response of Microcirculatory Oscillations to Hypoxia

The FMSF technique used with the PORH test is uniquely suitable for measuring the reaction of microcirculatory oscillations to hypoxia. This is especially important for understanding the function of the microcirculation in diseases associated with ischemia (hypoxia), including diabetes mellitus (DM). DM is a chronic, metabolic disease with numerous vascular complications. The most frequent are microvascular complications, including diabetic kidney disease (DKD) and diabetic foot ulceration (DFU). The latter complications are particularly challenging in terms of the unambiguous assessment of impaired microvascular function. Both these microvascular complications are directly linked to adaptive responses to hypoxia, which are mediated by the hypoxia-inducible factor (HIF) [47,48,49]. HIF-1α is directly responsible for the up-regulation of vascular endothelial growth factor (VEGF) expression. VEGF expression is necessary for the preservation of glomerular physiology. VEGF imbalance is associated with an increased risk of developing DKD [50]. VEGF is also an essential mediator of neovascularization, which is thought to be critical in diabetic wound healing [51].

**Figure 7:**
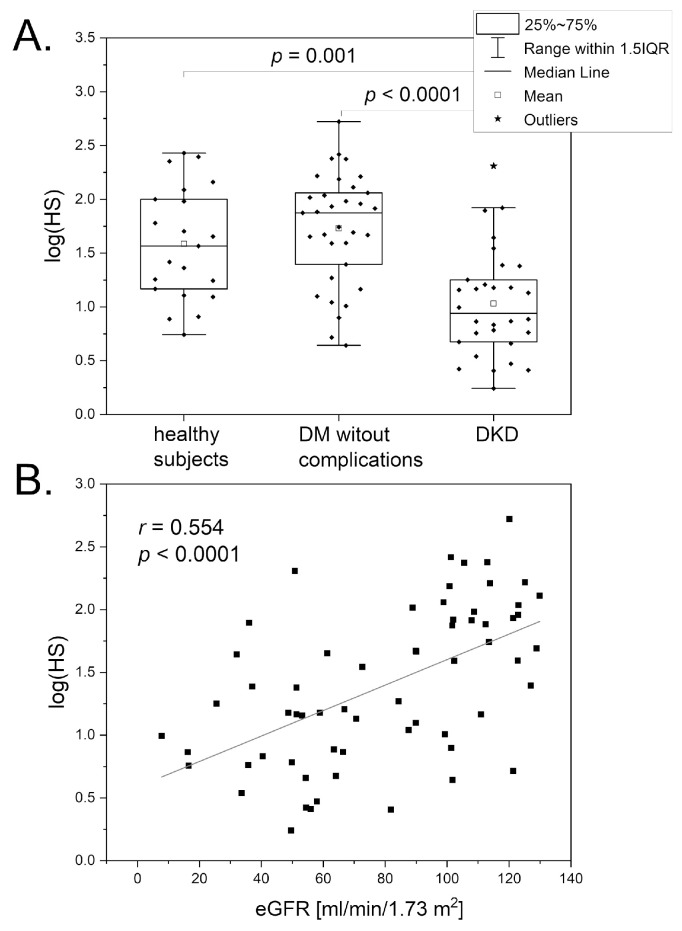
(**A**): A comparison of the log(HS) parameter in healthy individuals (n = 21, 8 males, 13 females, mean age 46.2 (30–68 y)), DM patients without complications (n = 33, 13 males, 20 females, mean age 44.4 (21–74 y)), and DKD patients (n = 30, 7 males, 23 females, mean age 62.8 (29–88 y)). (**B**): The correlation between the log(HS) parameter and nephrological parameter in the diabetic population (n = 63) (Pearson correlation). The differences between the parameters of the compared groups were considered statistically significant when *p* < 0.05. The *p*-values were calculated by one-way ANOVA with the Scheffe post hoc test. Reproduced from [52]. 2022, Elsevier B.V. RELX Group, Amsterdam, Netherlands.

The FMSF technique has been used to assess microvascular function in patients with DKD [52]. Both key FMSF parameters (RHR and HS) were found to have predictive potential. The HS parameter, describing the reaction of myogenic microcirculatory oscillations to transient hypoxia induced by the PORH test, seems well suited for assessing microvascular dysfunction in DKD. As shown in Figure 7A, the log(HS) parameter was significantly lower in the DKD group compared with the DM group without microvascular complications and also with the healthy group. The correlation between the log(HS) parameter and the estimated glomerular filtration rate (eGFR) is shown in Figure 7B. The log(HS) also negatively correlates with the advanced glycation end products (AGEs) [52]. These results clearly indicate that dysfunctional kidney function is associated with a low value for the HS parameter. Interestingly, patients with a very low value for the HS parameter (HS < 10) may also have impaired adaptation to physical exercise and high altitude.

The FMSF technique has also been used for the differentiation of diabetic foot ulcer (DFU), based on the stimulation of myogenic microcirculatory oscillations by transient ischemia [53]. It was found that a very low value for the HS parameter (HS < 5) can be used to predict DFU with a low prognosis of healing. These patients frequently also suffered from nephropathy, neuropathy, or prevalent cardiovascular disease. Additionally, they showed poor results in the USG Doppler test and had a significant history of amputation.

**Figure 8:**
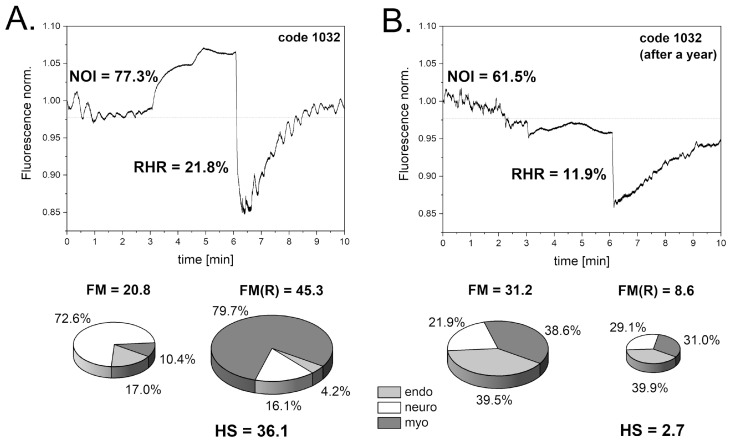
Significant changes in FMSF traces recorded at different time points for a patient with DFU (male, age 67 y, DM2). (**A**): First measurement. (**B**): Measurement performed after one year. Reproduced from [53]. 2021, Dove Medical Press Ltd. Publisher, Auckland, New Zealand.

As shown in Figure 8A, one patient with DFU had a satisfactory reaction to transient hypoxia, indicated by a moderate HS parameter value (HS = 36.1), suggesting potential DFU healing, though not guaranteed. A year later, the FMSF measurement showed a drastic decrease in the key FMSF parameters, indicating severe worsening of the DM disease (Figure 8B). The very low HS parameter value measured after a year (HS = 2.7) indicates the chronic status of DFU with a poor chance of healing. Observations of numerous cases of DFU patients with very low HS parameter values (HS < 5) showed no improvement in DFU status. This supports the conclusion that very low HS parameter values are indeed indicative of a poor prognosis for healing. This information is valuable for doctors in making decisions concerning further wound treatment.

## 5. Conclusions

**Figure 9:**
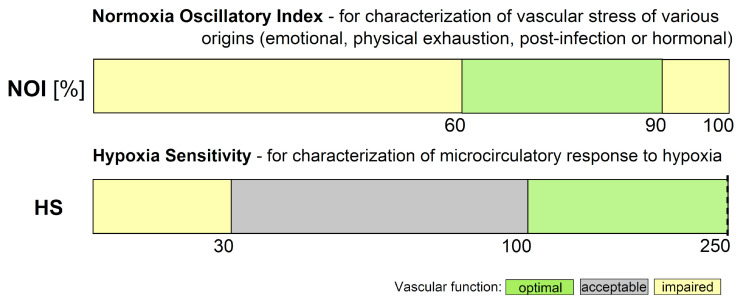
Ranges of FMSF parameters: NOI (normoxia oscillatory index) and HS (hypoxia sensitivity). Reproduced from [21]. 2024, MDPI AG Publisher, Basel, Switzerland.

Diagnosing microvascular circulation is crucial for preventive medicine practitioners, as it allows for the early identification of dysfunctional microvascular perfusion. However, there are very few diagnostic tools available for this purpose. The FMSF technique offers a powerful diagnostic tool for the characterization of microvascular circulation, based on oscillatory parameters measured under conditions of both normoxia (NOI parameter) and hypoxia (HS parameter). Based on the cases discussed in this review, ranges of values for these parameters have been identified corresponding to optimal, acceptable, or impaired microvascular function (Figure 9).

The FMSF technique can be used to evaluate microvascular perfusion and assess the risk of vascular-related complications, enabling earlier intervention and management strategies tailored to individual patient needs. Microcirculatory oscillations can be registered with high precision by the FMSF technique; therefore, this technique can provide a simple and non-invasive diagnostic tool for the characterization of microvascular function, with a wide variety of potential applications in preventive medicine. The FMSF technique is particularly attractive due to its non-invasive nature and the possibility of its adaptation for use in wearable devices.

The NADH/NAD^+^ redox balance monitored by the FMSF technique can also be used for characterization of the coupling between mitochondrial and vascular regulation. These aspects are presently being explored, and it is likely that the HS parameter can also be used for the characterization of mitochondrial function.
